# Reviewers for 2021

**DOI:** 10.4314/gmj.v55i4.1

**Published:** 2021-12

We are very grateful to all our reviewers for recommending manuscripts for publication. Our reviewers give of their time and expertise to the service of science. We recognise those who reviewed for us in 2021. We hope that many more will respond to our invitation in the coming years.

Abantanga, Francis: Ghana

Ablordey, Anthony: Ghana

Abokyi, Samuel: Ghana

Adam, Ahmed: South Africa

Adanikin, Abiodun: Nigeria

Addo, Edwina Beryl: Ghana

Addo, Kennedy: Ghana

Adedia, David: Ghana

Adeko, Oluseun: Nigeria

Adeniran, Abiodun: Nigeria

Adjei, Patrick: Ghana

Adokiya, Martin: Ghana

Adu-Aryee, Nii-Armah: Ghana

Afaa, Taiba: Ghana

Afriyie-Mensah, Jane: Ghana

Agbley, Daniel: Ghana

Agwu, Ezera: Uganda

Agyei-Nkansah, Adwoa: Ghana

Ahadzi, Dzifa: Ghana

Aheto, Justice Moses K.: Ghana

Aikins, Moses: Ghana

Akaba, Godwin: Nigeria

Akakpo, Patrick : Ghana

Akhiwu, Benjamin: Nigeria

Akpalu, Josephine: Ghana

Alagbe, Olayemi: Nigeria

Amissah-Arthur, Maame: Ghana

Amoakoh-Coleman, Mary: Ghana

Amoateng, Patrick: Ghana

Amoyaw-Asamoah, Isabella: Ghana

Anane-Fenin, Betty: Ghana

Andoh-Adjei, Francis-Xavier: Ghana

Ansong, Daniel: Ghana

Antwi, Sampson: Ghana

Anyanechi, Charles: Nigeria

Aryeetey, Richmond: Ghana

Asaleye, Christianah: Nigeria

Asare Quansah, Gloria: Ghana

Asare, Offei: Ghana

Asuquo, Marcus: Nigeria

Atta, Dzifa: Ghana

Awuku, Yaw: Ghana

Ayettey-Adamafio, Mary: Ghana

Ayisi-Boateng, Nana: Ghana

Baah, Winfred: Ghana

Badoe, Ebenezer: Ghana

Baidoo, Kenneth: Ghana

Balcioglu, Yasin Hasan: Turkey

Bello, Ajediran: Ghana

Beyuo, Vera: Ghana

Biritwum, Richard: Ghana

Boahen, Eric: Ghana

Boateng, Yaw: Ghana

Boima, Vincent: Ghana

Bonsu, Frank: Ghana

Boskabadi, Hassan: Iran

Cannegieter, Suzanne: Netherlands

Chernyshov, P. V.: Ukraine

Chichom-Mefire, Alain: Cameroon

Clegg-Lamptey, Joe Nat: Ghana

Dakubo, Jonathan: Ghana

Dalaba, Maxwell A.: Ghana

Darko, Kwame: Ghana

Dedey, Florence: Ghana

Der, Edmund: Ghana

Dogbe, Edith: Ghana

Donkor, Peter: Ghana

Dordoye, Eugene: Ghana

Duah, Amoako: Ghana

Dzefi-Tettey, Klenam: Ghana

Edwin, Frank: Ghana

Edzie, Emmanuel K. Mesi: Ghana

Ekem, Ivy: Ghana

Ekpe, Eyo: Nigeria

El-Gilany, Abdel-Hady: Egypt

Enimil, Anthony: Ghana

Essuman, Akye: Ghana

Etwire, Victor: Ghana

Fadare, Joseph: Nigeria

Fasina, Oluyemi: Nigeria

Feldman, Steven: United States

Fiagbe, Delali: Ghana

Fiakpornoo, Martina: Ghana

Fofie, Chris: Ghana

Forson, Audrey: Ghana

Gezawa, Ibrahim: Nigeria

Gumanga, Solomon: Ghana

Gupta, Amod: India

Gyedu, Adam: Ghana

Hesse, Afua: Ghana

Ho, Roger C.: Singapore

Hodgson, Abraham: Ghana

Idowu, Bukunmi: Nigeria

Igboeli, Nneka: Nigeria

Isara, Alphonsus: Nigeria

Issah, Kofi : Ghana

James, Peter: Australia

Johnston, Carolyn: United States

Kalu, Michael E.: Canada

Kelishadi, Roya: Ghana

Kenu, Ernest: Ghana

Kerr, Peter G: Australia

Khalifa, Ahmed: Egypt

Kilba, Charlyne: Ghana

Kitcher, Emmanuel: Ghana

Klufio, George: Ghana

Kokuro, Collins: Ghana

Koram, Kwadwo: Ghana

Kretchy, Irene: Ghana

Kwakyi, Edward: Ghana

Kyei, Mathew: Ghana

Lamptey, Roberta: Ghana

Langhammer, Birgitta: Norway

Larsen-Reindorf, Rita: Ghana

Lartey, Margaret: Ghana

Lee, Yew Kong: Malaysia

Lentoor, Antonio: South Africa

Lim, Rosemary H.: United Kingdom

Maged, Ahmed M.: Egypt

Mahmoudabadi, Ali Zarei: Ghana

Malm, Keziah: Ghana

Mante, Sunny: Ghana

Martinez, Edson Zangiacomi: Brazil

Mate-Kole, Charles: Ghana

Mektrirat, Raktham: Thailand

Mensah, Samuel: Ghana

Mensah, Yaw: Ghana

Meysamie, Alipasha: Iran

Michael, Godpower: Nigeria

Mishra, Jiten: India

Mohd Noor, Noor Haslina: Malaysia

Morhe, Emmanuel: Ghana

Morjaria, Priya: United Kingdom

Motilewa, Olugbemi: Nigeria

Mould-Millman, Nee-Kofi: United States

Mume, Celestine: Nigeria

Mustapha, Shettima : Nigeria

Mutalub, Yahkub: Nigeria

Mutambirwa, Shingai: South Africa

Nartey, Edmund: Ghana

Nasseri, Karim: Iran

Ndlovu, Kwazi: South Africa

Newman, Mercy: Ghana

Nguah, Samuel: Ghana

Nkyekyer, Kobina: Ghana

Nsaful, Josephine: Ghana

Nsaful, Kwesi: Ghana

Nsiah, Kwabena: Ghana

Ntsiea, Mokgobadibe: South Africa

Nuertey, Benjamin: Ghana

Obadeji, Adetunji: Nigeria

Obadeji, Adetunji: Nigeria

Obiri-Yeboah, Dorcas: Ghana

Odeniyi, Ifedayo: Nigeria

Odugbemi, Babatunde: Nigeria

Ofori-Adjei, David: Ghana

Ogunbode, Adetola: Nigeria

Ogunnubi , Oluseun: Nigeria

Ohene, Sally-Ann: Ghana

Okonkwo, Ogugua: Nigeria

Okyere, Perditer: Ghana

Olagunju, Andrew: Nigeria

Olasode, Olayinka: Nigeria

Ologe, Foluwasayo: Nigeria

Olowookere, Samuel: Nigeria

Oppong, Samuel: Ghana

Ossai, Edmund Ndudi: Nigeria

Owusu, Enid: Ghana

Owusu, Michael: Ghana

Owusu-Agyei, Seth: Ghana

Page, Andrew: Australia

Palmieri, Patrick: Peru

Peltonen, Markku: Finland

Phillips, Richard: Ghana

Platonov, Pyotr: Sweden

Prazeres, Filipe: Portugal

Quarde, Akuffo: United States

Quashie, Neils B.: Ghana

Quayson, Solomon: Ghana

Reich, Adam: Poland

Renner, Lorna: Ghana

Sagoe-Moses, Isabella: Ghana

Saheeb, Birch: Nigeria

Samba, Ali: Ghana

Sarfo, Fred: Ghana

Seneadza, Nana: Ghana

Shaki, Omna: India

Shenoy, Suchitra: India

Shittu, Akeem: Nigeria

Souza, Dziedzom K. De: Ghana

Tachi, Kenneth: Ghana

Tagbor, Harry: Ghana

Takure, Augustine: Nigeria

Technau, Karl-Gunter: South Africa

Tettey, Mark: Ghana

Torpey, Kwame: Ghana

Tuoyire, Derek: Ghana

Udofia, Emilia: Ghana

Ulzen-Appiah, Kofi: Ghana

Umeora, O.U.J.: Nigeria

Umuerri, Ejiroghene: Nigeria

Wang, Leyi: United States

Whiting, Susan J.: Canada

Yahaya, James: Uganda

Yang, Hongmei: United States

Yawson, Alfred: Ghana

Yeboah-Manu, Dorothy: Ghana

Yorke, Ernest: Ghana

Zewde, Yared: Ethiopia

**Editorial Office**

**Ghana Medical Journal**

editor@ghanamedj.org

